# Treating lower extremity malperfusion syndrome in acute type A aortic dissection with endovascular revascularization followed by delayed aortic repair

**DOI:** 10.1016/j.xjon.2022.02.017

**Published:** 2022-02-23

**Authors:** Elizabeth L. Norton, Felix Orelaru, Aroma Naeem, Xiaoting Wu, Karen M. Kim, David M. Williams, Shinichi Fukuhara, Himanshu J. Patel, G. Michael Deeb, Bo Yang

**Affiliations:** a Division of Cardiothoracic Surgery, Department of Surgery, Emory University, Atlanta, Ga; b Department of General Surgery, St Joseph Mercy, Ann Arbor, Mich; c Department of Cardiac Surgery, Michigan Medicine, Ann Arbor, Mich; d Department of Radiology, Michigan Medicine, Ann Arbor, Mich

**Keywords:** lower extremity, malperfusion syndrome, aortic dissection, endovascular, open aortic repair, ARDS, acute respiratory distress syndrome, ATAAD, acute type A aortic dissection, CK, creatine kinase, LE, lower extremity, LE-MPS, lower extremity malperfusion syndrome, MPS, malperfusion syndrome, non-MPS, No malperfusion syndrome

## Abstract

**Objective:**

To assess the outcomes of emergency revascularization with endovascular fenestration/stenting followed by delayed open aortic repair in patients with acute type A aortic dissection with lower extremity (LE) malperfusion syndrome (MPS); that is, necrosis and dysfunction of the lower extremity.

**Methods:**

From 1996 to 2019, among 760 consecutive acute type A aortic dissection patients 512 patients had no malperfusion syndrome (Non-MPS), whereas 26 patients had LE-MPS with/without renal MPS and underwent endovascular fenestration/stenting, open aortic repair, or both. Patients with coronary, cerebral, mesenteric, and celiac MPS, or managed with thoracic endovascular aortic repair, were excluded (n = 222). All patients with LE-MPS underwent upfront endovascular fenestration/stenting except 1 patient (with signs of rupture) who initially underwent emergency open aortic repair.

**Results:**

Among the LE-MPS patients, 17 (65%) had LE pain, 15 (58%) had abnormal motor function with 8 (31%) having paralysis, 10 (38%) had LE pallor, 17 (65%) had LE paresthesia, and 20 (77%) had LE pulselessness. Of the 25 patients undergoing upfront endovascular fenestration/stenting, 16 went on to open aortic repair, 3 survived to discharge without aortic repair, and 6 died before aortic repair (3-aortic rupture and 3-organ failure). In-hospital mortality among all patients was significantly higher in the LE-MPS group (31% vs 6.3%; *P* = .0003). Among those undergoing open aortic repair, postoperative outcomes were similar between groups, including operative mortality (18% vs 6.5%; *P* = .10). LE-MPS was a significant risk factor for in-hospital mortality (odds ratio, 6.0 [1.9, 19]; *P* = .002).

**Conclusions:**

In acute type A aortic dissection, LE-MPS was associated with high in-hospital mortality. Emergency revascularization with endovascular fenestration/stenting followed by delayed open aortic repair may be a reasonable approach.

## Graphical abstract

Summary of the study showing in-hospital mortality among all acute type A aortic dissection patients was significantly higher in the lower extremity malperfusion syndrome group versus no malperfusion syndrome group (31% [8 out of 26] vs 6.3% [32 out of 512]; *P* = .0003). Lower extremity malperfusion syndrome was defined as necrosis of the tissue of lower extremity and related organ failure.

**Figure:**
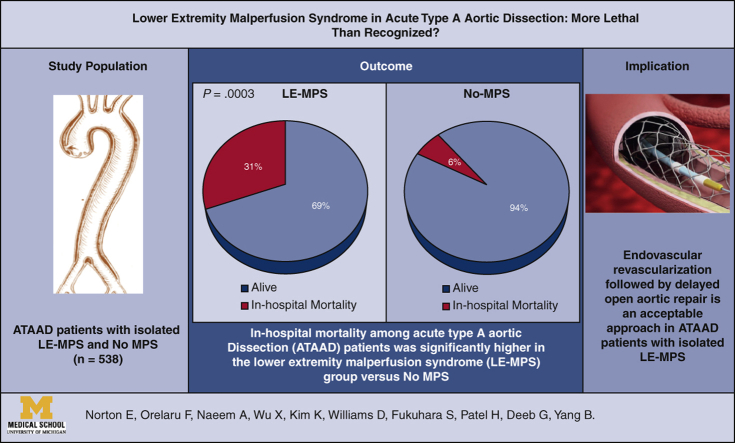

**Figure:**
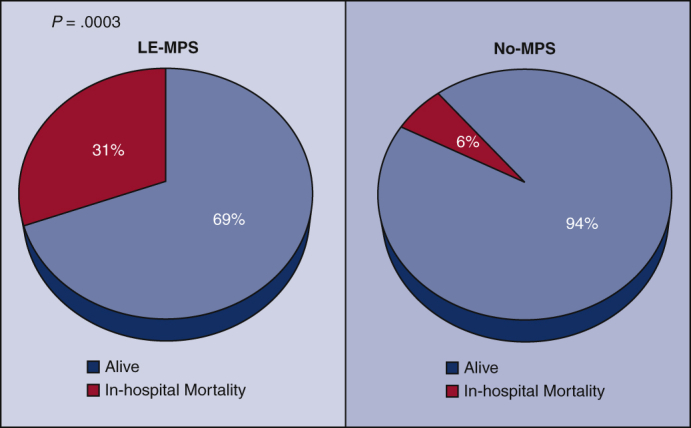
ATAAD patients with or without LE-MPS (necrosis and dysfunction of LE).

**Central Message**

Endovascular revascularization followed by delayed aortic repair had acceptable outcomes in ATAAD patients with lower extremity malperfusion syndrome (necrosis and dysfunction of the lower extremity).

**Perspective**

In-hospital mortality was high amongst patients with lower extremity malperfusion syndrome (necrosis and dysfunction of the lower extremity) in acute type A aortic dissection. Emergency revascularization with endovascular fenestration/stenting followed by delayed open aortic repair had acceptable surgical outcomes and may be a reasonable approach for this disease.

See Commentary on page 111.

Lower extremity (LE) malperfusion occurs in 15% to 40% of acute type A aortic dissection (ATAAD) cases.^1^ Prolonged malperfusion can result in malperfusion syndrome (MPS).^1^ MPS is a late-stage of malperfusion characterized by tissue necrosis and end-organ dysfunction due to dissection-related aortic branch vessel obstruction and insufficient blood flow to end organs.^2^ Likewise, LE-MPS is defined as inadequate blood flow with resultant LE tissue necrosis and sensory and motor dysfunction. Patients with ATAAD with concomitant MPS have a high perioperative mortality between 29% and 89%.^3^^,^^4^ The optimal surgical management for LE-MPS remains controversial. The conventional management of ATAAD with or without MPS is emergency open aortic repair.^5^^,^^6^ At the University of Michigan, we have treated LE-MPS patients with initial endovascular revascularization followed by delayed open proximal aortic repair due to multiorgan failure, which could significantly increase operative mortality of upfront emergency open aortic repair.^2^^,^^7^^,^^8^ In patients with MPS, the most critical life-threatening issue influencing outcomes is organ malperfusion, rather than aortic rupture.^2^^,^^7^ This study aimed to assess the outcomes of emergency revascularization with endovascular fenestration/stenting followed by delayed open aortic repair in ATAAD patients with LE-MPS.

## Methods

This study was approved by the Institutional Review Board at Michigan Medicine (HUM 001118517), a waiver of informed consent was obtained, and it was in compliance with Health Insurance Portability and Accountability Act regulations.

### Data Collection

Data from 1996 to 2019 was retrieved from the ATAAD registry at Michigan Medicine and supplemented with data from the Society of Thoracic Surgeons Michigan Medicine Cardiac Surgery Data Warehouse to identify the study cohort and determine pre-, intra-, and postoperative characteristics. These data were further supplemented with a retrospective medical record review. Information about survival was collected from the National Death Index Database through June 30, 2020.^9^

LE-MPS was diagnosed by clinical symptoms, including pulselessness, pain, motor or sensory deficit of the lower extremity; abnormal lab values (ie, elevated lactate, creatine kinase [CK], CKMB, and myoglobin) indicating tissue ischemia and necrosis; and radiographic evidence (computed tomography angiogram) of dynamic or static obstruction of arterial flow to the lower extremities. All patients with LE-MPS were confirmed to have muscle tissue necrosis from malperfusion, including serology (eg, CK, CKMB, myoglobin, and lactate) and clinical exam. Hemodynamically stable patients with LE-MPS underwent upfront endovascular fenestration/stenting before open aortic repair. Patients were then allowed to recover from lactic acidosis, shock, rhabdomyolysis, fasciotomy or amputation if needed, and acute respiratory distress syndrome (ARDS) before open proximal aortic repair.

Our technique for endovascular fenestration/stenting has been previously described.^3^^,^^10^^,^^11^ This technique includes angiographic evaluation of the various vascular territories, including the LE and subsequent fenestration of the dissection flap with a 16-mm diameter balloon, aortic true lumen stenting with a 16- to 18-mm diameter self-expanding stent if the true lumen remains collapsed, and/or branch vessel fenestration/stenting if a gradient >15 mm Hg persists between the aortic root or ascending aorta and a branch vessel.^12^ Details of endovascular intervention, including levels of aortic fenestration was detailed in Table E1.

### Patient Selection

**Figure 1:**
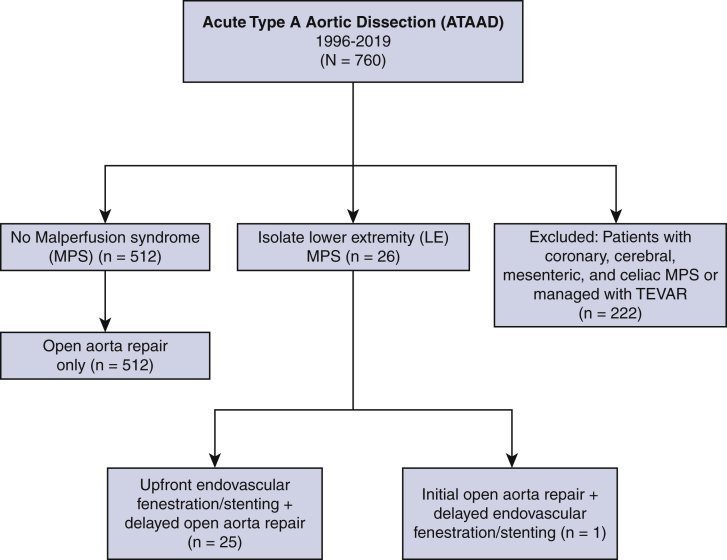
Consort diagram of selection and distribution of study population.

Between August 1996 and August 2019, a total of 760 patients presented with an ATAAD at our institution. Five hundred and twelve of those patients had no malperfusion syndrome (non-MPS) whereas 26 patients had LE-MPS with or without renal MPS and underwent endovascular fenestration/stenting, open aortic repair, or both. Patients with coronary, cerebral, mesenteric, and celiac MPS or managed with thoracic endovascular aortic repair were excluded (n = 222). All patients with LE-MPS underwent upfront endovascular fenestration/stenting except 1 patient (with signs of rupture) who initially underwent emergency open aortic repair. All patients in the non-MPS group underwent open aortic repair only (Figure 1). Patients with LE malperfusion (not MPS) were managed with emergency open aortic repair and included in the non-MPS group.

### Statistical Analysis

Data are presented as median (25%, 75%) for continuous data and n (%) for categorical data. Univariate comparisons between the groups were performed using Wilcoxon rank-sum tests for continuous data and χ^2^ tests for categorical data. Logistic regression models were used to calculate the odds ratio (OR) of significant factors for in-hospital mortality adjusting age, sex, cardiogenic shock, acute renal failure, renal MPS, and LE-MPS. These variables were chosen based on their clinical relevance and our previous studies.^7^^,^^11^ Due to small sample size, a Firth correction model was performed. The Kaplan-Meier method with log-rank testing was used to describe survival over time. Statistical calculations were performed using SAS version 9.4 (SAS Institute Inc).

## Results

### Preoperative Demographic Data

Compared with the non-MPS group, the LE-MPS group had a significantly higher proportion of acute renal failure (42% vs 3.5%), renal malperfusion (31% vs 0%), and spinal cord malperfusion (7.7% vs 0%). The median time from admission to open aortic repair was longer in the LE-MPS group compared with the non-MPS group (1 vs 0 days; *P* < .0001). Otherwise, preoperative comorbidities were similar between LE-MPS and non-MPS groups (Table 1).

**Table 1:** Demographic and preoperative characteristics of all patients

Characteristic	All patients	LE-MPS	Non-MPS	*P* value
	(N = 538)	(n = 26)	(n = 512)	
Admission variables				
Age on admission (y)	60 (50-69)	62.5 (51-71)	60 (50-69)	.44
BMI	28.2 (24.7-32)	30.2 (24.7-32.3)	28.1 (24.7-32)	.74
Male sex	359 (67)	17 (65)	342 (67)	.88
CAD	96 (18)	3 (12)	93 (19)	.45
History of MI	30 (5.6)	2 (7.7)	28 (5.5)	.66
Previous cardiac intervention	85 (16)	5 (19)	80 (16)	.58
Previous cardiac surgery	42 (7.8)	4 (15)	38 (7.4)	.14
Hypertension	386 (72)	22 (85)	364 (71)	.14
COPD	55 (10)	4 (16)	51 (10)	.31
Smoking status				.28
Never smoker	233 (43)	10 (38)	223 (44)	
Former smoker	148 (27)	5 (19)	143 (28)	
Current smoker	155 (29)	11 (42)	144 (28)	
Diabetes	36 (6.7)	1 (4.0)	35 (6.8)	1.0
Creatinine on admission (mg/dL)	1.0 (0.8-1.2)	1.0 (0.9-1.6)	1.0 (0.8-1.2)	.058
Creatinine clearance (mL/min)	90.5 (68.5-120.0)	73.0 (52.3-112.9)	91.0 (69.8-120.5)	**.04**
Chronic kidney disease	16 (3.0)	1 (3.9)	15 (2.9)	.55
History of CVA	20 (3.7)	0 (0)	20 (3.9)	.62
PVD	85 (16)	6 (23)	79 (15)	.28
Connective tissue disorder	27 (5.0)	1 (3.9)	26 (5.1)	1.0
Ejection fraction (%)	55 (55-60)	55 (53-65)	55 (55-60)	.90
Aortic insufficiency				.68
None	136 (27)	6 (24)	130 (27)	
Trace/trivial	55 (11)	5 (20)	50 (10)	
Mild	112 (22)	5 (20)	107 (22)	
Moderate	87 (17)	4 (16)	83 (17)	
Severe	118 (23)	5 (20)	113 (23)	
Cardiogenic shock	43 (8.0)	1 (3.9)	42 (8.2)	.71
Acute stroke	2 (0.4)	0 (0)	2 (0.4)	1.0
Acute MI	0 (0)	0 (0)	0 (0)	
Acute renal failure	29 (5.4)	11 (42)	18 (3.5)	**<.0001**
Malperfusion syndrome				
Spinal cord malperfusion	2 (0.4)	2 (7.7)	0 (0)	**.002**
Renal malperfusion	8 (1.5)	8 (31)	0 (0)	**<.0001**
Management				
IR	26 (4.8)	26 (100)	0 (0)	**<.0001**
Time from admission to IR (d)	NA	0 (0, 1)	NA	NA
Open aortic repair	529 (98)	17 (65)	512 (100)	**<.0001**
Time from admission to aortic repair (d)	0 (0-1)	1 (1-3)	0 (0-1)	**<.0001**
Time from IR to aortic repair (d)	NA	1 (1-2.5)	NA	NA

Values are presented as median (interquartile range) for continuous variables and number (%) for categorical variables. *P* value <.05 is statistically significant. *BMI*, Body mass index; *CAD*, coronary artery disease; *MI*, myocardial infarction; *COPD*, chronic obstructive pulmonary disease; *CVA*, cerebrovascular accident; *PVD*, peripheral vascular disease; *IR*, endovascular procedure by interventional radiology; *NA*, not applicable.

### LE-MPS

Among the patients in the LE-MPS group, 17 (65%) had LE pain, 15 (58%) had abnormal motor function with 8 (31%) having paralysis, 10 (38%) had LE pallor, 17 (65%) had LE paresthesia, and 20 (77%) had LE pulselessness. One patient with LE-MPS had signs of aortic rupture and initially underwent emergency open aortic repair followed by endovascular fenestration/stenting. The other 25 patients underwent upfront endovascular fenestration/stenting. Sixty-four percent (16 out of 25) of patients had open aortic repair, whereas 24% (6 out of 25) died before aortic repair (3 aortic rupture and 3 organ failure) (see Table 2). Additionally, 3 patients survived to discharge without aortic repair for the following reasons: lack of patient's interest in proceeding with open aortic repair, previous aortic root and ascending aorta replacement, and poor surgical candidacy for open repair. Those 3 patients have all survived for more than 2 years after discharge. Maximum serum lactate level was significantly higher in patients who died due to aortic rupture or organ failure compared with patients who survived endovascular fenestration/stenting (6.0 mmol/L vs 2.0 mmol/L; *P* = .02) (see Table 2).

**Table 2:** Clinical condition of patients with lower extremity malperfusion syndrome based on the outcome of endovascular reperfusion

Condition	Death	Survival∗	*P* value†
	(n = 6)	(n = 20)	
Age on admission (y)	70 (61-80)	60 (50-69.5)	.10
Male sex	3 (50)	14 (70)	.63
CAD	1 (17)	2 (10)	.65
History of MI	1 (17)	1 (5.0)	.42
Previous cardiac surgery	1 (17)	3 (15)	1.0
Hypertension	4 (67)	18 (90)	.22
COPD	1 (17)	3 (15)	1.0
Diabetes	0 (0)	1 (5)	1.0
Smoking history	4 (67)	12 (60)	1.0
Creatinine on admission (mg/dL)	0.9 (0.7-1.5)	1.1 (1.0-1.7)	.22
Chronic kidney disease	0 (0)	1 (5)	1.0
PVD	0 (0)	6 (30)	.28
Cardiogenic shock	0 (0)	1 (5)	1.0
Acute renal failure	3 (50)	8 (40)	1.0
Spinal cord malperfusion	1 (17)	1 (5)	.42
Renal malperfusion	2 (33)	6 (30)	1.0
Max creatinine before OR/death/discharge	1.9 (0.8-2.8)	1.4 (1.1-2.0)	.84
Max serum lactate before OR/death/discharge (mmol/L)	6.0 (4.1-8.4)	2.0 (1.8-4.0)	**.02**
pH before OR/death/discharge	7.3 (7.2-7.3)	7.3 (7.2-7.4)	.14
Max CK before OR/death/discharge	5485 (529-32,456)	1533 (305-11,428)	.63
Max CKMB before OR/death/discharge	24 (9.3-217)	18.7 (4.5-25.8)	.59
Requiring fasciotomy	2 (33)	4 (20)	.60
Requiring amputation	0 (0)	0 (0)	–
Admit to IR (h)	3.0 (2.8-3.5)	3.6 (3.0-4.8)	.32
Length of IR (h)	3.8 (2.6-6.0)	5.0 (3.4-5.9)	.59

*P* value <.05 is statistically significant. *CAD*, Coronary artery disease; *MI*, myocardial infarction; *COPD*, chronic obstructive pulmonary disease; *PVD*, peripheral vascular disease; *OR*, open aortic repair; *CK*, creatine kinase; *CKMB*, creatine kinase-MB; *IR*, endovascular procedure by interventional radiology.

∗ Patients survived to open aortic repair or discharge without open repair.

† *P* value indicates the difference between the groups of death from organ failure and survival to open aortic repair or hospital discharge.

### Postprocedure/Operative Outcomes

Among all patients, the LE-MPS group had significantly higher in-hospital mortality after endovascular fenestration/stenting or open aortic repair (31% vs 6.3%; *P* = .0003), but other postintervention outcomes, including atrial fibrillation, new-onset renal failure, paraplegia, among others were similar between groups (Table 3).

**Table 3:** Outcomes after interventional radiology (IR) or open aortic repair (OR) outcomes of patients with lower extremity malperfusion syndrome (LE-MPS) or nonmalperfusion syndrome (non-MPS)

Outcome	All patients (N = 538)	LE-MPS (n = 26)	Non-MPS (n = 512)	*P* value
Reoperation for bleeding	41 (7.6)	2 (7.7)	39 (7.6)	1.0
Tamponade	9 (1.7)	0 (0)	9 (1.8)	1.0
Postoperative MI	6 (1.1)	1 (3.9)	5 (1.0)	.26
Atrial fibrillation	178 (33)	8 (31)	170 (33)	.80
New-onset CVA	35 (6.5)	0 (0)	35 (6.8)	.40
New-onset paraplegia	1 (0.2)	0 (0)	1 (0.2)	1.0
Sepsis	8 (1.5)	1 (3.9)	7 (1.4)	.33
Pneumonia	79 (15)	5 (19)	74 (14)	.57
Reintubation	33 (6.1)	3 (12)	30 (5.9)	.21
Tracheostomy	15 (2.8)	2 (7.7)	13 (2.5)	.16
New-onset acute renal failure	56 (10)	3 (12)	53 (10)	.74
Requiring new dialysis	21 (3.9)	0 (0)	21 (4.1)	.62
Total LOS (d)	10 (7, 16)	12 (6, 24)	10 (7, 16)	.52
In-hospital mortality	40 (7.4)	8 (31)	32 (6.3)	**.0003**

In the LE-MPS group, any complications after IR procedures or OR were recorded as outcomes. In the non-MPS group, any complications after OR were recorded as outcomes. Values are presented as median (interquartile range) for continuous variables and number/total number (%) for categorical variables. *P* value <.05 is statistically significant. *MI*, Myocardial infarction; *CVA*, cerebrovascular accident; *LOS*, length of stay.

Patients with LE-MPS who successfully underwent initial endovascular stenting/fenestration followed by an open aortic repair had significantly longer postoperative lengths of stay compared with non-MPS patients (14 vs 10 days; *P* = .047). Otherwise, there were no significant differences in outcomes, including new-onset paraplegia, stroke, in-hospital mortality, and operative mortality, between groups after open aortic repair (Table 4). Among the LE-MPS group, 6 patients (23%) underwent an LE fasciotomy and 0 patients underwent LE amputation. Among all ATAAD patients (both LE-MPS and non-MPS), LE-MPS was a significant risk factor for in-hospital mortality (OR, 6.0; 95% CI 1.9-19; *P* = .002) as was cardiogenic shock (OR, 5.2; 95% CI, 2.3-12.1; *P* = .0001) (Table 5).

**Table 4:** Postoperative outcomes of patients with or without lower extremity malperfusion syndrome (LE-MPS) (only patients who underwent open aortic repair)

Outcome	All patients (N = 529)	LE-MPS (n = 17)	Non-MPS (n = 512)	*P* value
Reoperation for bleeding	9 (1.8)	0 (0)	9 (1.8)	1.0
Tamponade	9 (1.7)	0 (0)	9 (1.8)	1.0
Perioperative MI	5 (1.0)	0 (0)	5 (1.0)	1.0
Atrial fibrillation	177 (33)	7 (41)	170 (33)	.49
DSWI	12 (2.3)	0 (0)	12 (2.3)	1.0
Sepsis	8 (1.5)	1 (5.9)	7 (1.4)	.23
New-onset CVA	35 (6.6)	0 (0)	35 (6.8)	.62
New-onset paraplegia	1 (0.2)	0 (0)	1 (0.2)	1.0
Pneumonia	79 (15)	5 (29)	74 (14)	.15
Reintubation	33 (6.2)	3 (18)	30 (5.9)	.08
Tracheostomy	15 (2.8)	2 (12)	13 (2.5)	.08
Postoperative AKI	55 (10)	2 (12)	53 (10)	.69
Requiring new dialysis	21 (4.0)	0 (0)	21 (4.1)	1.0
Postoperative LOS (d)	10 (7, 15)	14 (9, 24)	10 (7, 15)	**.047**
Intraoperative mortality	5 (1.0)	0 (0)	5 (1.0)	1.0
In-hospital mortality	34 (6.4)	2 (12)	32 (6.3)	.30
30-d mortality	28 (5.3)	2 (12)	26 (5.1)	.23
Operative mortality∗	36 (6.8)	3 (18)	33 (6.5)	.10

Values are presented as median (interquartile range) for continuous variables and number/total number (%) for categorical variables. *P* value <.05 is statistically significant. *Non-MPS*, No malperfusion syndrome; *MI*, myocardial infarction; *DSWI*, deep sternal wound infection; *CVA*, cerebrovascular accident; *AKI*, acute kidney injury; *LOS*, length of stay.

∗ Defined as in-hospital mortality or mortality within 30 days after open repair.

**Table 5:** Firth correction model for risk factors of in-hospital mortality

Variables	Odds ratio (95% CI)	*P* value
LE-MPS	6.0 (1.9-19)	**.0024**
Age	1.0 (0.99-1.05)	.20
Male sex	1.8 (0.8-3.9)	.14
Acute renal failure	2.9 (0.9-9.4)	.07
Concomitant renal MPS	0.7 (0.1-5.1)	.68
Cardiogenic shock	5.2 (2.3-12.1)	**.0001**

*P* value <.05 is statistically significant. *LE-MPS*, Lower extremity malperfusion syndrome; *MPS*, malperfusion syndrome.

### Long-Term Outcomes

**Figure 2:**
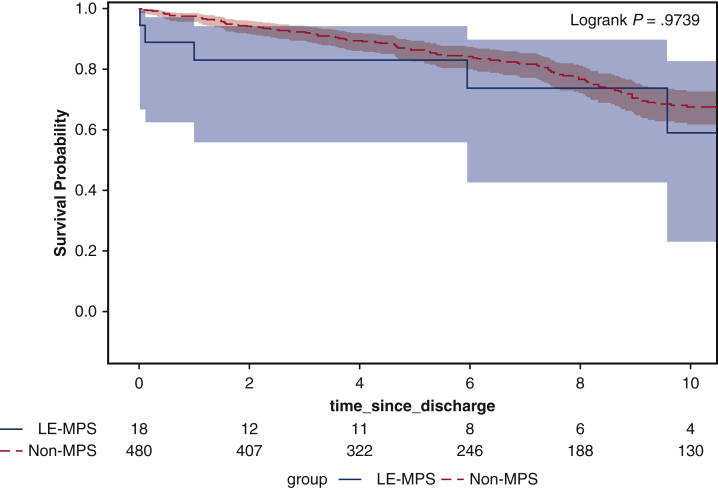
Kaplan-Meier analysis showed the long-term survival was not significantly different between patients with acute type A aortic dissection (ATAAD) with lower extremity malperfusion syndrome (MPS) and no MPS groups (10-year, 59%; 95% CI, 23%-83% vs 68%; 95% CI, 62%-73%; *P* = .97). *LE-MPS*, Lower extremity malperfusion syndrome; *No-MPS*, no malperfusion syndrome.

The median follow-up time was 6.3 years. The completeness to follow-up was 100%. In the patients who were discharged from the hospital, there was no significant difference in long-term survival between ATAAD patients with LE-MPS and non-MPS groups (10-year, 59%; 95% CI, 23%-83% vs 68%; 95% CI, 62%-73%; *P* = .97) (Figure 2).

## Discussion

**Figure 3:**
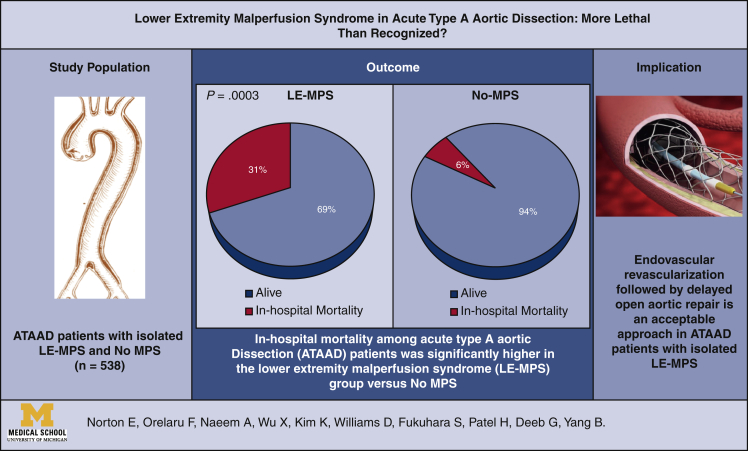
Summary of the study showing in-hospital mortality among all acute type A aortic dissection patients was significantly higher in the lower extremity malperfusion syndrome (*LE-MPS*) group versus non-MPS group (31% [8 out of 26] vs 6.3% [32 out of 512]; *P* = .0003). LE-MPS was defined as necrosis of the tissue of LE and related organ failure. *No-MPS*, No malperfusion syndrome; *ATAAD*, acute type A aortic dissection.

In this study, the patients with LE-MPS had a significantly higher overall in-hospital mortality (31%) compared with patients without LE-MPS (6%). In patients with LE-MPS who were treated with emergency LE revascularization and recovered from MPS, postoperative outcomes and long-term survival were similar to the patients without LE-MPS (Figure 3, Video Abstract and Video 1).

There has been a confusion of malperfusion and MPS in the literature. We define malperfusion as compromised blood flow to end organs, the cause of MPS, and MPS is the consequence of prolonged malperfusion; that is, the necrosis and dysfunction of the end organs from end organ malperfusion,^7^^,^^13^ and in this specific study, the end-organ was the LEs. MPS frequently is complicated with multiorgan failure and metabolic acidosis. The difference between malperfusion and MPS is similar to the difference of “bacteremia and sepsis (septic syndrome) or “HIV and AIDS.”^14^ Malperfusion of the LE was not an indication for emergency endovascular fenestration/stenting and delayed open aortic repair, such as loss of femoral artery pulse but with normal function of the LE. However, MPS was an indication for emergency endovascular fenestration/stenting, such as loss of femoral pulse with LE motor or sensory deficit, elevated CK or serum lactate level, and radiographic evidence of dynamic or static obstruction of iliac or femoral arteries. All 26 patients with LE-MPS in our study had clinical evidence of LE malperfusion and subsequent necrosis and dysfunction of LE.

For ATAAD patients with LE malperfusion, we all are in agreement that those patients should be treated with emergency open aortic repair. However, for ATAAD patients with LE-MPS (necrosis and dysfunction of LE), same as mesenteric MPS, the optimal management remains controversial. The conventional wisdom is still an emergency open aortic repair to resolve LE malperfusion and prevent aortic rupture.^5^^,^^6^ At the University of Michigan, we have treated patients with LE-MPS with initial endovascular revascularization followed by delayed open proximal aortic repair due to extremely high operative mortality (80%-90%) of emergency open aortic repair in patients with preoperative MPS.^2^^,^^7^^,^^8^ We believe that in those patients, expeditious open aortic repair can resolve only dynamic malperfusion of the LE but cannot resolve MPS (ie, necrosis of the LE that has already happened in patients) and its complications, such as organ failure and metabolic acidosis. Instead, upfront emergency aortic repair can worsen the LE-MPS due to the persistent static malperfusion to the LE during the open aortic repair and massive inflammatory reaction of the body to cardiopulmonary bypass and hypothermic circulatory arrest. A recent study from the Cleveland Clinic showed that 30% of patients with LE malperfusion need additional revascularization for ongoing extremity ischemia after open aortic repair for ATAAD.^15^ The open aortic repair only resolved lower extremity malperfusion in 70% of the patients. Endovascular fenestration/stenting can resolve both static and dynamic LE malperfusion.^7^^,^^13^ During endovascular fenestration/stenting, we measured the blood pressure in the femoral artery and ascending aorta to confirm the LE malperfusion was resolved for every patient. Because of the necrosis of LE, those patients could quickly develop multiorgan failure after resolution of malperfusion due to ischemia/reperfusion injury, namely acute renal failure (42% in patients with LE-MPS in this study), ARDS, severe metabolic acidosis, and hyperkalemia that could result in arrhythmia and asystole. Therefore, we recommend delayed open aortic repair only after patients recovery from metabolic acidosis and multiorgan failure (namely ARDS), and blood CK levels start decreasing, indicating no ongoing necrosis of the LE; likely when these patients can tolerate cardiopulmonary bypass and hypothermic circulatory arrest with a low risk of being on extracorporeal membrane oxygenation postoperatively. In patients with MPS and the metabolic derangements associated with it, an endovascular procedure to correct the malperfusion is much more tolerable than an open aortic operation on cardiopulmonary bypass. Endovascular fenestration/stenting resolves the malperfusion with minimal surgical trauma to salvage the living/borderline tissue in the leg as much as possible. After reperfusion, the limb could be preserved and the patient can recover. However, if a patient has an obviously dead leg due to prolonged malperfusion, amputation should be performed.

Delayed open aortic repair in ATAAD patients after upfront endovascular revascularization could place patients at risk of aortic rupture.^7^ In this study, the median time from admission to open aortic repair was 24 hours longer in the LE-MPS group compared with the non-MPS group. Six patients died before open aortic surgery. Three of them died from organ failure after all malperfusion was resolved with fenestration/stenting, which achieved similar results as open aortic repair but with much less trauma and influence on those patients. The other 3 patients died from aortic rupture that could have been prevented by open aortic repair (Table E2). The maximum serum lactate level was 6 mmol/L and CK level was >5000, indicating severe ischemia and necrosis of the lower extremity. Their operative mortality would be 33% to 89%.^3^^,^^15^ In this whole cohort, 3out of 25 (12%) patients died from aortic rupture, which was still much lower than the operative mortality of emergency open aortic repair. Most of our aortic ruptures happened during the first decade. During the second decade, as we gained more experience of medically managing ATAAD patients, only 4% of patients had aortic rupture in all patients we managed with upfront fenestration/stenting.^7^ The risk of dying from multiorgan failure in patients with MPS is 6 times higher than dying from aortic rupture.^7^ Nevertheless, the aortic rupture was higher in this cohort of patients with LE-MPS compared with mesenteric malperfusion syndrome.^11^ We should be more cautious for patients with isolated LE-MPS with or without renal malperfusion and repair the dissected proximal aorta in those patients whenever we think the patients can tolerate cardiopulmonary bypass and circulatory arrest without being on extracorporeal membrane oxygenation postoperatively.

Our in-hospital mortality of ATAAD patients with LE-MPS was comparable to the study from the Cleveland Clinic with an in-hospital mortality of 33% in ATAAD patients who underwent emergency open aortic repair and subsequent revascularization of the LE.^15^ The open aortic repair in those patients did not resolve LE malperfusion, but likely had much more influence on the patients than endovascular fenestration/stenting. Other studies have shown lower 30-day mortality^16^ or lower OR (OR, 2) of peripheral malperfusion for 30-day mortality using Nordic consortium scoring.^17^ Most likely those discrepancies were due to the different patient populations included. Those studies included patients with malperfusion and MPS, and we included patients with only LE-MPS (ie, tissue necrosis and dysfunction of the LE). Other studies used 30-day mortality after the operation, whereas we used in-hospital mortality, which included any death after 30 days from the operation. Other studies did not include deaths of patients who did not have an operation due to poor surgical candidacy caused by multiorgan failure from LE malperfusion. Our study included all deaths in patients with LE malperfusion with or without an open aortic repair.

All studies that treated LE-MPS with emergency open aortic repair report significantly higher postoperative rate of permanent strokes (25%), acute renal failure (38%), fasciotomy (50%), sepsis, gastrointestinal or pulmonary complications, and lengths of stay in LE malperfusion patients compared with patients without LE malperfusion.^2^^,^^15^^,^^16^ In our study, preoperatively, the LE-MPS group had significantly higher proportion of acute renal failure compared with the non-MPS group. But among the majority of patients (76%) who survived LE-MPS and undergoing open aortic repair, outcomes including postoperative strokes, renal failure, sepsis, and pneumonia, among others were similar between groups. Although some of those could be due to type II error because the sample size was small in the LE-MPS group, our findings indicated that emergency upfront endovascular revascularization improved postoperative outcomes, especially renal failure requiring hemodialysis and permanent strokes. Long-term survival of patients with LE-MPS following discharge from the hospital alive was similar to the patients without LE-MPS. Taken together, our strategy of treating patients with ATAAD with emergency endovascular revascularization by fenestration/stenting followed by delayed open aortic repair produced acceptable perioperative and long-term outcomes.

This study has limitations as a single center, retrospective study. There was no control group of LE-MPS patients treated with immediate open aortic repair. The International Registry of Acute Aortic Dissection (IRAD) data were not used as control group due to the varying definitions of malperfusion and MPS and the inability to distinguish a uniform control group. Our study was designed as a descriptive study to report the outcomes of ATAAD patients with LE-MPS treated with endovascular fenestration/stenting followed by delayed open aortic repair. The sample size was small and could yield type II error.

## Conclusions

Outcomes were favorable in stable ATAAD patients with LE-MPS treated with emergency revascularization via endovascular fenestration/stenting followed by delayed open aortic repair. Our strategy in this sick patient population may be a reasonable approach.

### Conflict of Interest Statement

The authors reported no conflicts of interest.

The *Journal* policy requires editors and reviewers to disclose conflicts of interest and to decline handling or reviewing manuscripts for which they may have a conflict of interest. The editors and reviewers of this article have no conflicts of interest.

## Figures and Tables

**Table E1:** Details of endovascular interventions (26∗ patients treated for lower extremity malperfusion syndrome)

Level of aortic fenestration/stenting	Aortic fenestration	Aortic stenting	Branch vessel thrombolysis/thromboembolectomy	Branch vessel fenestration	Branch Vessel Stenting
Descending thoracic	0	1	–	–	–
Supraceliac	4	2	–	–	–
Celiac	0	0	0	0	0
Supramesenteric	8	8	–	–	–
Mesenteric	1	0	0	0	0
Suprarenal	1	1	–	–	–
Renal	1	0	0	0	3
Infrarenal	12	17	–	–	–
Iliac	–	–	7	1	19

∗ One patient presented with signs of aortic rupture, cardiac tamponade, cardiogenic shock, and right lower extremity malperfusion and went to immediate open repair.

**Table E2:** Detailed cause of death in patients following endovascular fenestration/stenting by interventional radiology, but before open repair or discharge

Case	Age	Year of treatment	Cause of death
1	74	2005	Respiratory failure, family withdrew care
2	61	2007	Rupture
3	50	2014	Rupture
4	84	2016	Arrythmia; withdrawal of care
5	80	2016	Renal failure, extremity ischemia, respiratory failure; family withdrew care
6	66	2017	Rupture

## Supplementary Data

Video 1 Discussion of the influence of upfront endovascular revascularization on survival outcomes in acute type-A dissection patients with lower extremity malperfusion syndrome. Video available at: https://www.jtcvs.org/article/S2666-2736(22)00078-X/fulltext.

Video Abstract Video available at: https://www.jtcvs.org/article/S2666-2736(22)00078-X/fulltext.
