# Supplementary figures and images for: Treating lower extremity malperfusion syndrome in acute type A aortic dissection with endovascular revascularization followed by delayed aortic repair
Source: JTCVS Open. 2022 Feb 23;10:101–10. doi: 10.1016/j.xjon.2022.02.017 (PMC9667713; doi:10.1016/j.xjon.2022.02.017)

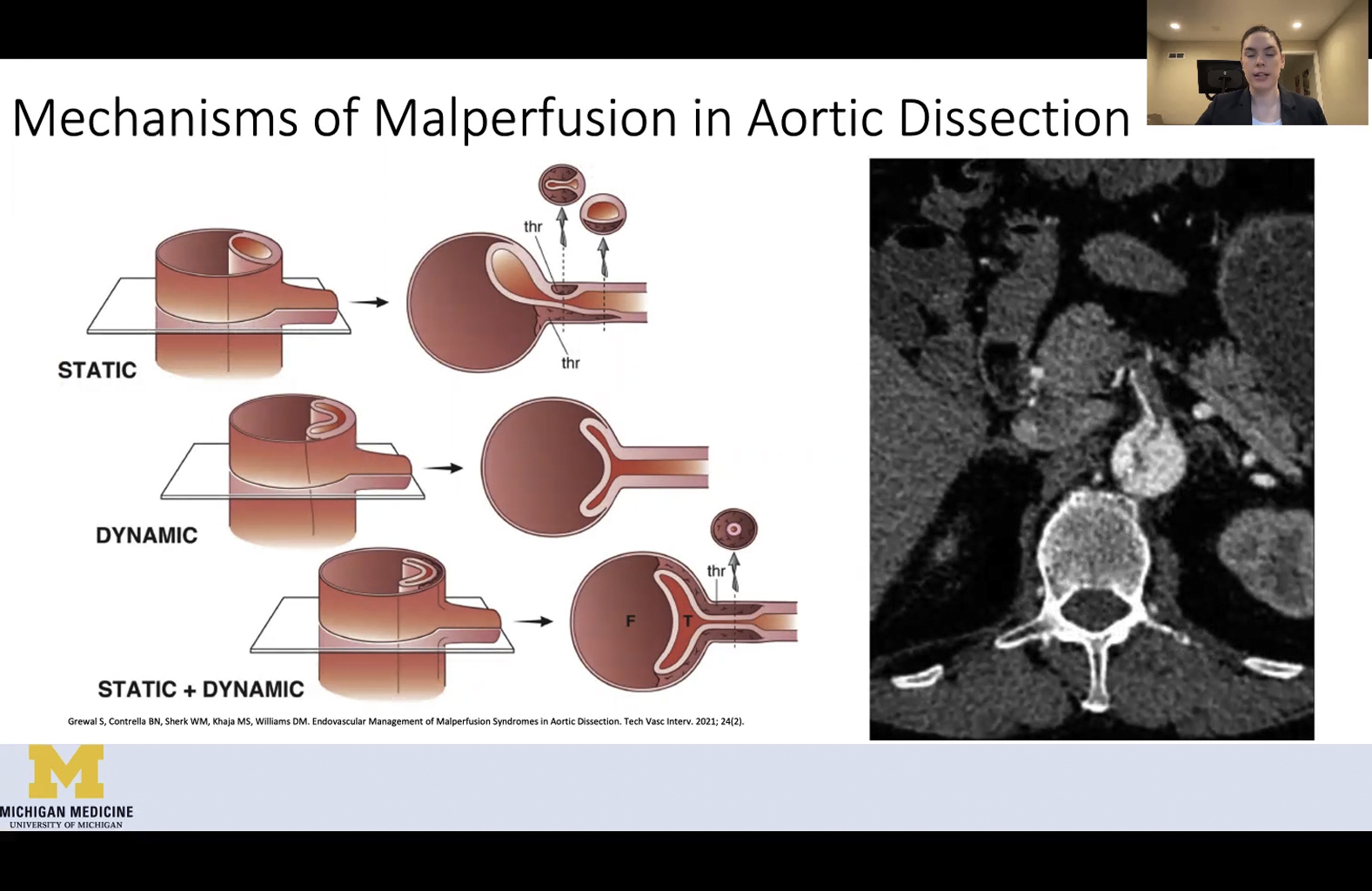

Supplement: Video 1 — Discussion of the influence of upfront endovascular revascularization on survival outcomes in acute type-A dissection patients with lower extremity malperfusion syndrome. Video available at: https://www.jtcvs.org/article/S2666-2736(22)00078-X/fulltext. [file fx3.jpg]
